# Mapping and Characterization of the *fefe* Gene That Controls Iron Uptake in Melon (*Cucumis melo* L.)

**DOI:** 10.3389/fpls.2017.01003

**Published:** 2017-06-14

**Authors:** Raghuprakash Kastoori Ramamurthy, Brian M. Waters

**Affiliations:** Department of Agronomy and Horticulture, University of Nebraska–Lincoln, LincolnNE, United States

**Keywords:** iron uptake and metabolism, bHLH transcription factor, mutant proteins, *Cucumis melo*, gene expression regulation

## Abstract

Iron (Fe) deficiency in plants limits crop growth and productivity. Molecular mechanisms that plants use to sense and respond to Fe deficiency by coordinated expression of Fe-uptake genes are not fully understood. The C940-fe chlorotic melon (*Cucumis melo*) mutant known as *fefe* is unable to upregulate Fe-uptake genes, however, the *FeFe* gene had not been identified. In this study, we used two F_2_ mapping populations to map and identify the *FeFe* gene as *bHLH38*, a homolog of subgroup Ib *bHLH* genes from *Arabidopsis thaliana* that are involved in transcriptional regulation of Fe-uptake genes in partnership with the *FIT* gene. A Ty1-copia type retrotransposon insertion of 5.056 kb within *bHLH38* is responsible for the defect in *bHLH38* in *fefe*, based on sequencing and expression analysis. This retrotransposon insertion results in multiple non-functional transcripts expected to result in an altered and truncated protein sequence. Hairy root transformation of *fefe* plants using wild-type *bHLH38* resulted in functional complementation of the chlorotic *fefe* phenotype. Using a yeast-2-hybrid assay, the transcription factor Fit interacted with the wild-type bHLH38 protein, but did not interact with the *fefe* bHLH38 protein, suggesting that heterodimer formation of Fit/bHLH38 to regulate Fe-uptake genes does not occur in *fefe* roots. The second subgroup Ib *bHLH* gene in the melon genome is not functionally redundant to *bHLH38*, in contrast to Arabidopsis where four subgroup Ib *bHLH* genes are functionally redundant. Whereas the Arabidopsis *bHLH* transcript levels are upregulated by Fe deficiency, melon *bHLH38* was not regulated at the transcript level. Thus, the *fefe* mutant may provide a platform for studying *bHLH38* genes and proteins from other plant species.

## Introduction

Iron (Fe) is crucial for plant growth, development, and productivity (Briat et al., 2015). Iron is involved in chlorophyll synthesis and is a constituent of certain enzymes involved in metabolism (Kobayashi and Nishizawa, 2012; Bashir and Nishizawa, 2013). Iron deficiency is a major limiting factor for crop production, especially in alkaline soils, which occur on approximately 30% of the earth (Chen and Barak, 1982). Plant species can be classified into two categories based on their Fe uptake mechanisms (Marschner et al., 1986). Iron uptake in graminaceous species, known as Strategy II, is characterized by production of high-affinity Fe(III) binding compounds called phytosiderophores, which are secreted into the rhizosphere to form phytosiderophore-Fe(III) complexes. These complexes are taken up by the root cells through a specific plasma membrane transport system (Römheld and Marschner, 1986; Curie et al., 2001). Iron uptake in dicotyledonous and non-graminaceous monocots, known as Strategy I, is characterized by soil acidification by H^+^-ATPase proteins, reduction of Fe(III) to Fe(II) by ferric chelate reductase (FCR) proteins and subsequent uptake of Fe(II) by Fe transporter proteins (Kobayashi and Nishizawa, 2012). In *Arabidopsis thaliana, H^+^-ATPase* 2 (*AHA2*) encodes the proton extrusion component (Santi and Schmidt, 2009). The primary root surface FCR is encoded by *Ferric Reduction Oxidase 2* (*FRO2*) (Robinson et al., 1999), and analogous genes in cucumber (*Cucumis sativus* L.) and melon (*C. melo* L.) are called *FRO1* (Waters et al., 2007, 2014). Fe(II) transporters include *IRT1* (Varotto et al., 2002; Vert et al., 2002) and *Nramp1* (Curie et al., 2000; Castaings et al., 2016).

Strategy I Fe-uptake genes are regulated largely at the transcriptional level. *AtFRO2, AtIRT1, AtNRAMP1* and various other genes are transcriptionally activated by the basic helix-loop-helix (bHLH) transcription factor AtFit1 (Colangelo and Guerinot, 2004; Yuan et al., 2005). The subgroup Ib genes of the bHLH superfamily, *bHLH38, bHLH39, bHLH100* and *bHLH101*, are upregulated by Fe deficiency in Arabidopsis (Wang et al., 2007; Dinneny et al., 2008; Buckhout et al., 2009; Yang et al., 2010; Bauer and Blondet, 2011; Schuler et al., 2011; Stein and Waters, 2012; Waters et al., 2012; Andriankaja et al., 2014; Maurer et al., 2014). The AtFit1 protein interacts with these subgroup Ib bHLH proteins to regulate Fe-uptake genes as a heterodimer complex (Yuan et al., 2008; Wang et al., 2013). The *bHLH38, bHLH39, bHLH100* and *bHLH101* genes are functionally redundant in Arabidopsis, as single, double, or triple loss-of-function *bHLH* mutations do not have a chlorotic Fe deficiency phenotype under Fe sufficient conditions (Wang et al., 2007; Sivitz et al., 2012; Andriankaja et al., 2014; Maurer et al., 2014), except in one report (Wang et al., 2013).

The chlorotic melon C940-Fe *(fefe*) mutant originated spontaneously in the melon cultivar Edisto (Nugent and Bhella, 1988). The genetic basis for *fefe* was retained by outcrossing the original mutant plant to the cultivar Mainstream and self-pollinating chlorotic mutants until the F_5_ generation, resulting in the C940-fe germplasm (Nugent, 1994). The *fefe* mutant plants are incapable of inducing Strategy I Fe-uptake responses (Von Jolley et al., 1991; Welkie, 1996), and Fe-uptake gene expression (Waters et al., 2014; Hsieh and Waters, 2016), and cannot survive under normal culture conditions unless it is supplemented with Fe, similar to *FIT* mutants in Arabidopsis. 82 genes, including Fe-uptake genes and riboflavin synthesis genes, were not regulated by Fe-deficiency in *fefe* plants compared to their WT counterpart (Waters et al., 2014; Hsieh and Waters, 2016), suggesting that the *fefe* gene could be a transcription factor. Since the *fefe* lesion is not in the melon *FIT* gene, *FeFe* was predicted to act upstream of *FIT* or as a partner of Fit (Waters et al., 2014). The main objective of this work is to map and characterize the *fefe* gene. We used genetic, genomic, transcriptomic and molecular approaches to map and functionally characterize the *fefe* gene. The results of this research will provide increased understanding of Fe-homeostasis in Strategy I plant species.

## Materials and Methods

### Genetic Mapping

An F_2_ mapping population consisted of 269 individuals from a cross between “snake melon” (PI 435288) and C940-fe. The population was genotyped and scored, and the chlorosis trait associated with the *fefe* mutation was mapped to an 8 cM region of linkage group 8 (LG8) (Ramamurthy and Waters, 2015). To fine map the *fefe* mutation, a second, 288 individual F_2_ mapping population was developed from a cross between “pocket melon” (PI 536481) and C940-fe. This F_2_ mapping population was grown in hydroponics as described below prior to scoring leaf chlorosis in F_2_ plants as “0” (chlorotic) or “1” (normal). The normal and chlorotic *fefe* mutant F_2_ plants were distinguishable 1–2 weeks after planting in nutrient solution. DNA was isolated from a single young leaf from each plant (Kang et al., 1998), and quantity and purity of DNA was assessed using spectrophotometry at 260 and 280 nm. DNA was diluted to 25 ng/μl and stored at -20°C until genotyping.

A total of 112 SSR markers for LG8 from the consensus genetic map (Diaz et al., 2011) and markers for LG8 provided by Syngenta on the ICUGI website^1^ were tested, and in total, 27 markers were polymorphic and were used for genotyping the F_2_ mapping population (**Table 1**). PCR reactions were performed in a final volume of 10 μl with 1×*Taq* buffer [(16 mM (NH_4_)_2_SO_4_, 67 mM Tris–HCL (pH 8.8 at 25°C), 0.1% stabilizer], 2 mM MgCl_2_, 0.15 mM dNTP, 1 μM each primer, 0.2 U *Taq* DNA polymerase (Bioline USA Inc., Taunton, MA, United States), and 20 ng DNA. The cycling conditions were: an initial cycle at 94°C for 3 min, followed by 40 cycles at 94°C, 30 s, 55–58°C, 30 s and 72°C, 30 s, and a final extension step at 72°C, 5 min. PCR products were visualized with UV light after electrophoresis in 3% superfine resolution agarose (Amresco LLC, Solon, OH, United States) gels with 1× TBE (0.9 M Tris-borate, 0.002 M EDTA, pH 8.0), stained with ethidium bromide.

**Table 1:** Linkage mapping of the *fefe* gene using two different F_2_ mapping populations and a joint linkage map.

F2-population	N^a^	LG	Number of markers	Map length (cM)	Position of peak (cM)	Linked marker	LOD	PVE^b^
Snake melon X *fefe* population (I)	269	8	14	173	81	CMN038	53.5	59
Pocket melon X *fefe* population (II)	288	8	27	96	79	CMACC146	62.4	63
Joint map (I+II)	557	8	35	121	81	CMN038	115.5	62

*^a^N- Number of individuals, ^b^PVE- Percentage of phenotypic variation explained by each locus.*

Phenotype scores for 288 pocket melon × C940-fe F_2_ individuals and their corresponding genotypes across 27 loci in population were entered into a spreadsheet as an input file for QTL analysis in R/qtl software (Broman et al., 2003). Genetic positions were deduced for markers that were not present in the consensus map (from Syngenta and Li et al., 2011). Data checking steps for genotyping were performed using standard R/qtl functions (Broman et al., 2003). To obtain a better resolution of the *fefe* locus, a joint linkage map consisting of 557 individuals in both snake melon X *fefe* and pocket melon X *fefe* populations was constructed using polymorphic markers (*N* = 35) on LG8. For joint map construction, genotypic (LG8) and phenotypic information from the two mapping populations was input for QTL analysis in R/qtl software (Broman et al., 2003). The genetic maps for individual and joint analysis were constructed using est.rf and est.map functions of R/qtl. Interval mapping was performed using the “scanone” function which detects a single QTL by performing genome wide scan with possible allowance for covariates, with a binary model to analyze the binary phenotype (i.e., *fefe* or *FeFe*).

### Growing Condition of Plants Used for RNA-seq

Seeds were imbibed in germination paper soaked with 0.2 mM CaSO_4_ for 4 days, then were transferred to hydroponic containers. Seedlings were placed in sponge holders in lids of black plastic containers, four plants per 750 ml solution, with continuous aeration. The nutrient solution had the following composition: 0.8 mM KNO_3_, 0.4 mM Ca(NO_3_)_2_, 0.3 mM NH_4_H_2_PO_4_, 0.2 mM MgSO_4_, 25 μM CaCl_2_, 25 μM H_3_BO_3_, 2 μM MnCl_2_, 2 μM ZnSO_4_, 0.5 μM CuSO_4_, 0.5 μM Na_2_MoO_4_, 100 mM MES buffer (pH 5.5) and 10 μM Sprint 138 (Becker-Underwood, Ames, IA, United States). Plants were grown in a growth chamber with lighting provided by a mixture of incandescent and fluorescent sources at 250 μmol m^-2^ s^-1^ for a photoperiod of 16 h (on at 06:00 and off at 22:00). For the +/- Fe RNA-seq experiment, Edisto and *fefe* mutants were pretreated for 9 days on -Cu solution, and *fefe* mutants that had green leaves were used for treatments of 3 days duration in -Fe nutrient solution or 20 μM Sprint 138. The purpose for the -Cu pretreatment was to use only healthy *fefe* plants [since the *fefe* chlorotic phenotype can be rescued using -Cu treatment (Waters et al., 2014)].

### RNA-seq and Differential Expression Analysis

Total RNA was extracted from roots using the Plant RNeasy kit (Qiagen, Hilden, Germany). RNA quality and concentration was determined by UV spectrophotometry. Sources of RNA samples were as described in the previous section. RNA-seq was performed at the University of Nebraska Medical Center Next Generation Sequencing Core Facility using an Illumina HiSeq 2000 instrument. Barcoded libraries were constructed from 3 μg of root total RNA, with three biological replicate libraries per treatment. Replicates were run in separate lanes, with a total of six samples from different treatments in each lane. The short reads are available as NCBI BioProject: PRJNA371826^2^. Because melon and cucumber genomes are orthologous and the cucumber genome sequence and annotation is complete (Huang et al., 2009; González et al., 2010), the cucumber transcriptome was used as a reference for read mapping. Trimming of primers and adapters was performed using Trimmomatic (V0.32), read mapping was performed using BOWTIE2 (Langmead and Salzberg, 2012) with –local-N1 option, conversion of mapped reads into sam format was performed using SAMtools (Li et al., 2009) and extraction of read counts from sam files was performed using perl scripts, as previously reported (Waters et al., 2014). For gene expression analysis, the data matrix was imported into R and analyzed using the Bioconductor package DESeq (Anders and Huber, 2010). The count data was normalized for library size and then transformed using variance stabilization. Poisson distributions of normalized counts for each transcript were compared for different conditions using a negative binomial test. Differential expression was called for genes with a false discovery rate moderated *q*-value < 0.05 (Benjamini and Hochberg, 1995), and also showed a 1.0 log fold-change in expression and >10 reads in at least one treatment. *De novo* reconstruction of the *bHLH38* transcript was performed using Trinity software (V.r20131110) (Haas et al., 2013). IGVviewer 2.3^3^ was used to view the reads that are mapped onto the reference sequence as applicable.

### Reverse Transcription-PCR

One microgram of DNase treated RNA (RNase-free DNase I, New England Biolabs, Ipswich, MA, United States) from roots of -Fe and -Fe/-Cu treated Edisto and *fefe* and from eight normal and eight mutant snake melon X *fefe* F2 roots was used for cDNA synthesis, using the High Capacity cDNA Reverse Transcription kit (ABI, Foster City, CA, United States) with random hexamers at 2.5 μM final concentration. The cDNA templates were PCR amplified using primers spanning the insertion: *fefe*_mrkr_F-5′-AGAAACTGAGTAATCCGGCGA-3′ and *fefe*_mrkr_R-5′ TCGACTTGCAGAAATTATCGA-3′.

### Edisto *bHLH38* Cloning and Hairy Root Transformation

Edisto *bHLH38* (MELO3C019065) full length genomic sequence (2.334 kb promoter +2.32 kb gene) was PCR amplified using the primers *bHLH38*_Promoter_F 5′-TCCCTTTGAACCAATGATGG-3′ and *bHLH38*_XbaI_R 5′-GCATGATCTAGAACACATTGATATATATGGTTAATAA-3′. Phusion High-fidelity DNA polymerase (Thermo Scientific) was used for PCR amplification of *bHLH38* following manufacturer’s instructions. An *Xba*1 restriction site was present in the promoter region at position 379 bp of 2.334 kb, and an *Xba*I site was in the reverse primer, underlined above. After *Xba*I restriction (NEB Biolabs), the resulting bHLH38 *Xba*1 fragment was cloned into the pHairyRed (Lin et al., 2011) destination vector. The Edisto-*bHLH38* genomic construct was transformed into the K599 strain of *Agrobacterium rhizogenes* (generously provided by Dr. Christopher Taylor, The Ohio State University) using freeze-thaw transformation (Wise et al., 2006). K599 containing pHairyRed-Edisto *bHLH38* genomic fragment or K599 containing pHairyRed (empty vector) was grown in YEP plates with streptomycin and kanamycin selection. Agrobacteria suspension was prepared as previously described (Kereszt et al., 2007), and plug preparation and inoculation of Agrobacteria into rock wool plugs was performed as described (Chabaud et al., 2006), except we used hydroponic liquid media (described above) instead of half-strength MS. Stem sections with one or two axillary nodes from 1-month-old *fefe* plants were cut and inserted into the hole in the rock wool plug, and the plants were covered in a humid chamber for 4–5 days under ambient light. The humid chamber was opened for dehydration treatment for several hours until the leaves were not turgid, and the humid chamber was closed. Hairy roots developed 2–3 weeks after transformation. Transgenic roots were distinguished from non-transgenic roots, based on the presence of DsRed fluorescence. Z-series images were acquired on a Nikon A1+ CLSM mounted on a Nikon 90i compound microscope. Excitation of DsRed was at 561 nm and emission was detected at 575–625 nm. Image series were projected to form a single image. The transmitted light images were simultaneously acquired, but only a single image plane is presented. At least two biological experiments were performed to obtain the transgenic plants.

### Ferric-Chelate Reductase Activity

Root ferric reductase assays were performed for 50 min on transgenic roots (positive for DsRed fluorescence) and non-transgenic roots of *fefe* plants that were grown on -Fe solution for 2 days, using 20 ml of an assay solution. The assay solution was composed of 0.1 mM ferrozine (3-(2-pyridyl)-5,6-diphenyl-1,2,4-triazine-4′,4′-disulfonic acid sodium salt; Sigma–Aldrich), 0.1 mM Fe(III)-EDTA and 1 mM MES buffer (pH 5.5) (Fisher Scientific, Fair Lawn, NJ, United States). Change of assay solution from colorless to purple indicates ferric-chelate reductase activity.

### Yeast 2-Hybrid Interactions

The Matchmaker^TM^ GAL4 Two-Hybrid System 3 (Clontech) was used for the yeast 2-hybrid experiment. Coding sequences for Edisto *FIT*, Edisto *bHLH38* and the *fefe bHLH38* with its 14 bp insertion were amplified from cDNA and were cloned into pGEM-T vector and were later cloned as EcoRI fragment into pGADT7 and pGBKT7 yeast two-hybrid vectors. Drop assays were performed by growing AH109 transformants on synthetic dropout (SD) liquid media without leucine (L) and tryptophan (T) at 29°C until an OD_600_ of >0.5 is reached. Cultures were then diluted to an OD_600_ of 0.1 and diluted in a 10× dilution series. For each dilution, 5 μl of cell suspension was spotted on SD media without leucine, tryptophan, histidine (H) and adenine (A).

## Results

### *FeFe* Gene Mapping

**FIGURE 1:**
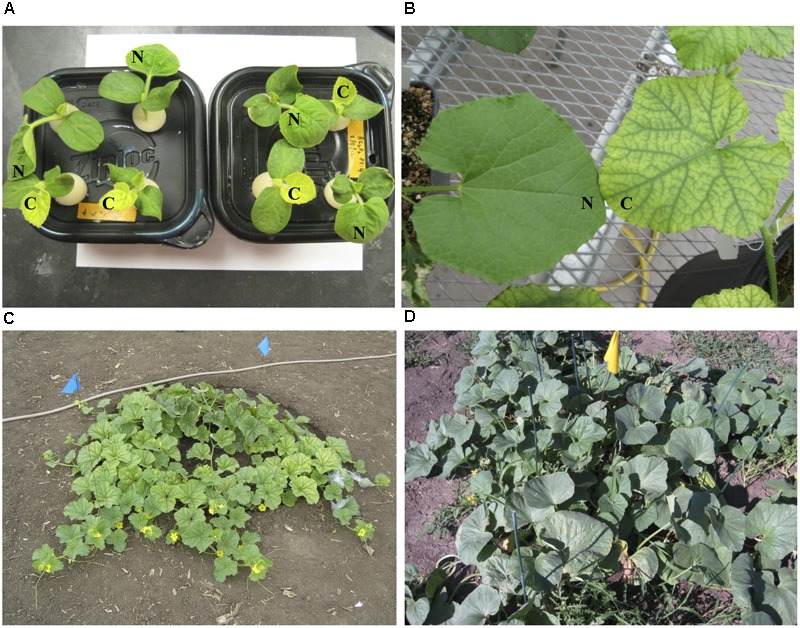
Leaf color phenotypes of *fefe* (chlorotic) and wild-type Edisto plants grown under different conditions in this study. **(A)** Pocket melon X *fefe* F_2_ plants grown in hydroponics. Scoring for chlorosis phenotype is indicated by N (normal) or C (chlorotic); **(B)** Greenhouse grown plants cultivated in commercial potting mix. **(C)** Field grown *fefe* mutant plant; **(D)** Field grown wild-type Edisto plant.

**FIGURE 2:**
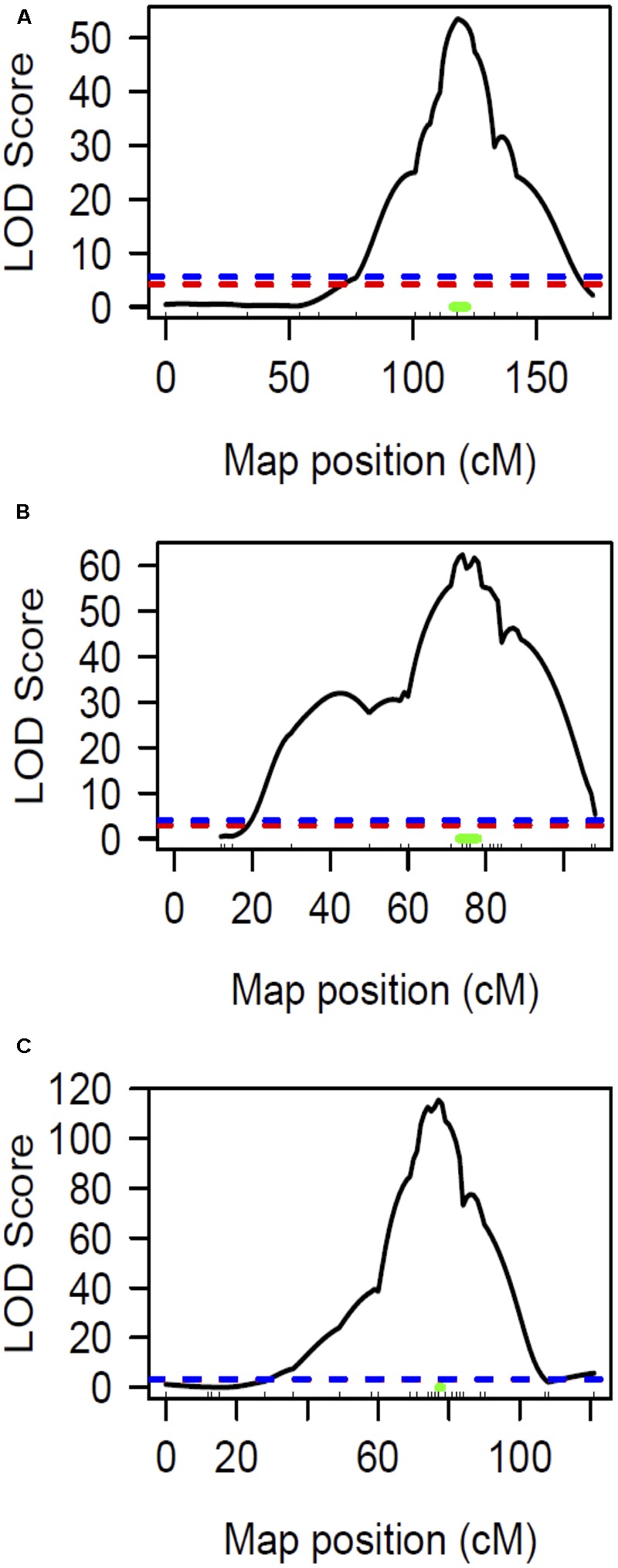
Linkage mapping of the *fefe* gene on chromosome 8. Ticks inside *X*-axis represent the position of markers. The blue and red dashed lines represent the permutation test specified LOD threshold at *p*-values of 0.05 and 0.01, respectively. The green bar represents Bayesian confidence interval associated with the *fefe* locus. **(A)** Interval mapping analysis using snake melon X *fefe* F_2_ (*n* = 269); **(B)** Interval mapping analysis using pocket melon X *fefe* F_2_ (*n* = 288); **(C)** Interval mapping analysis using joint linkage map (both populations) consisting of 557 F_2_ individuals.

The chlorotic phenotype (**Figure 1**) mapped to a single locus on LG8 using the snake melon X *fefe* F_2_ mapping population (Ramamurthy and Waters, 2015) (**Figure 2A**). Using Syngenta marker positions, the *fefe* locus was mapped to a 6 cM (2-LOD support) (**Figure 2A**) region in the snake melon X *fefe* population. The percentage of variation explained by LG8 locus was approximately 59% (**Figure 2A** and **Table 1**). Due to the low polymorphism rate of 12% in the snake melon X *fefe* population, we could not further narrow down the *fefe* genetic interval with the markers available. Therefore, we also mapped the chlorosis phenotype in a pocket melon X *fefe* F_2_ mapping population. Of 288 F_2_ plants, 196 had normal green leaves and 92 had chlorotic leaves. 27 markers on LG8 were polymorphic in the parents. The distribution of the parental genotypes in the F_2_ population was almost equal based on 27 polymorphic loci on LG8, with 20.7% pocket melon genotype, 30.1% C940-fe genotype and 42.2% heterozygous. The *fefe* locus was mapped to a 4 cM interval, with a LOD score of 62 (**Figure 2B** and **Table 1**), with 63% of the variation explained by the peak (**Table 1**). Since both populations in this study had a common parent, C940-fe (*fefe*), we used joint linkage analysis to refine the *fefe* genetic interval to a 1 cM peak on LG8 with a LOD score of 115 (**Figure 2C** and Supplementary Data S1A). The closest marker at the peak explained ∼61% of the variation (**Figure 2C** and **Table 1**).

### *FeFe* Candidate Genes

**FIGURE 3:**
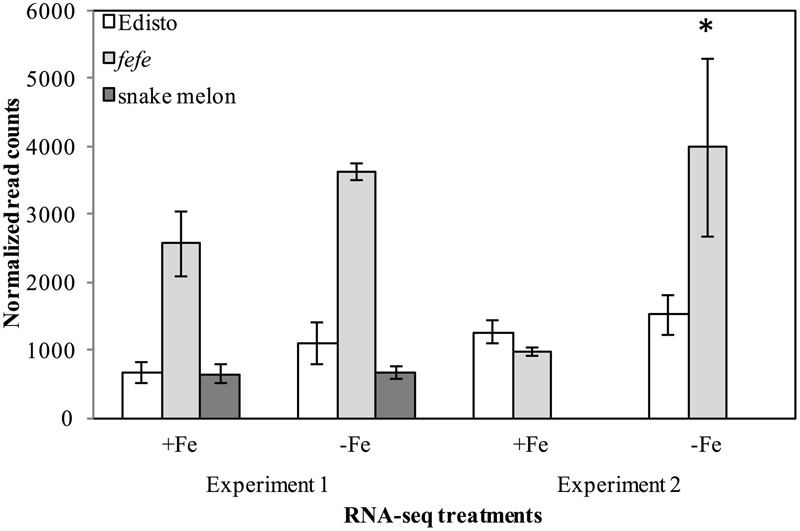
Gene expression (normalized read counts ± SD) of *bHLH38* in wild-types Edisto and snake melon and the *fefe* mutant under Fe deficient and Fe sufficient control conditions in two independent RNA-seq experiments. Experiment 1 is from Waters et al. (2014); experiment 2 is from this study. ^∗^Indicates significant difference between Fe-deficient and control treatment means at FDR < 0.05.

Since the *fefe* gene was predicted to be a transcription factor (Waters et al., 2014), we explored possible candidates within the 1 cM confidence interval. The genetic interval spanning the *fefe* gene, between the markers DM0766 and DM0640, corresponded to two scaffolds (scaffold0068 and scaffold0036) of the melon genome draft (Garcia-Mas et al., 2012). There were 186 predicted genes (Supplementary Data S1B) in this interval. There were six genes annotated as transcription factors in the mapped interval, based on homology to *Arabidopsis thaliana*: AT3G56970.1 *BHLH038*; AT5G04150.1 *BHLH101*; AT3G57390.2 *AGL18*; AT5G62470.1 *MYB96*; AT3G57040.1 *ARR9* (RESPONSE REGULATOR 9); and AT3G10760.1 myb family transcription factor. We ruled out *AGL18, MYB96* and *ARR9* as unlikely candidates for *fefe* based on their function (To et al., 2004; Verelst et al., 2007; Seo et al., 2011). The AT3G10760.1 myb family transcription factor is putatively involved in the fruit ripening process (Pillet et al., 2015). Two transcription factors in the interval, *bHLH38* and *bHLH101*, are associated with regulation of Fe-uptake genes (Wang et al., 2007). To determine if these genes were polymorphic between *fefe* and WT Edisto, or were differentially regulated, we sequenced the cDNAs and observed the normalized RNA-seq read counts from two independent experiments. Sequencing the *bHLH101* gene showed that it was not polymorphic between the mutant and WT plants. The expression of *bHLH101* was extremely low in both WT and mutant plants, ranging from 0 to 3 total raw read counts (compared to average read counts of approximately 450) under Fe replete or Fe-deficient conditions, suggesting that *bHLH101* can be considered not expressed. The *bHLH38* transcript levels were much higher than *bHLH101* both in WT and *fefe*, ranging from 965 to 3979 read counts under Fe replete or deficient conditions, respectively. Transcript abundance did not change significantly under Fe deficiency in Edisto and snake melon WT roots. Although the trend of Fe regulation appeared similar in WT and *fefe*, the *fefe bHLH38* expression differed in the two RNA-seq experiments (**Figure 3**). In the first experiment, *bHLH38* was not significantly up-regulated by Fe deficiency, but in the second experiment, *bHLH38* abundance was about fourfold higher under Fe deficiency due mainly to an unusually low read count in the +Fe *fefe* sample. We also checked abundance of bHLH38 transcript by RT-PCR (data not shown) and confirmed that the transcript is not regulated by Fe deficiency.

**FIGURE 4:**
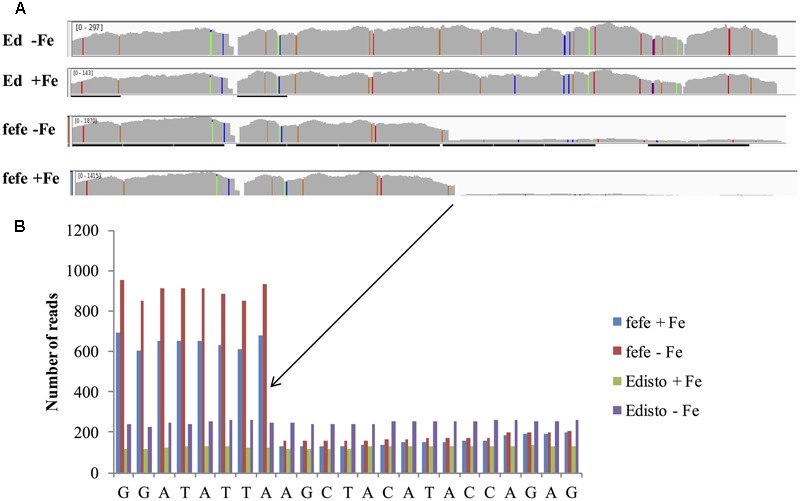
RNA-seq reads of melon *bHLH38* mapped onto cucumber reference transcript Csa4M434480.1. **(A)** Read mapping of *bHLH38* full length transcript in Edisto and *fefe* under Fe sufficient or deficient conditions; **(B)** Detail of sequence around site of the abrupt decrease in read counts in *fefe* bHLH38.

**FIGURE 5:**
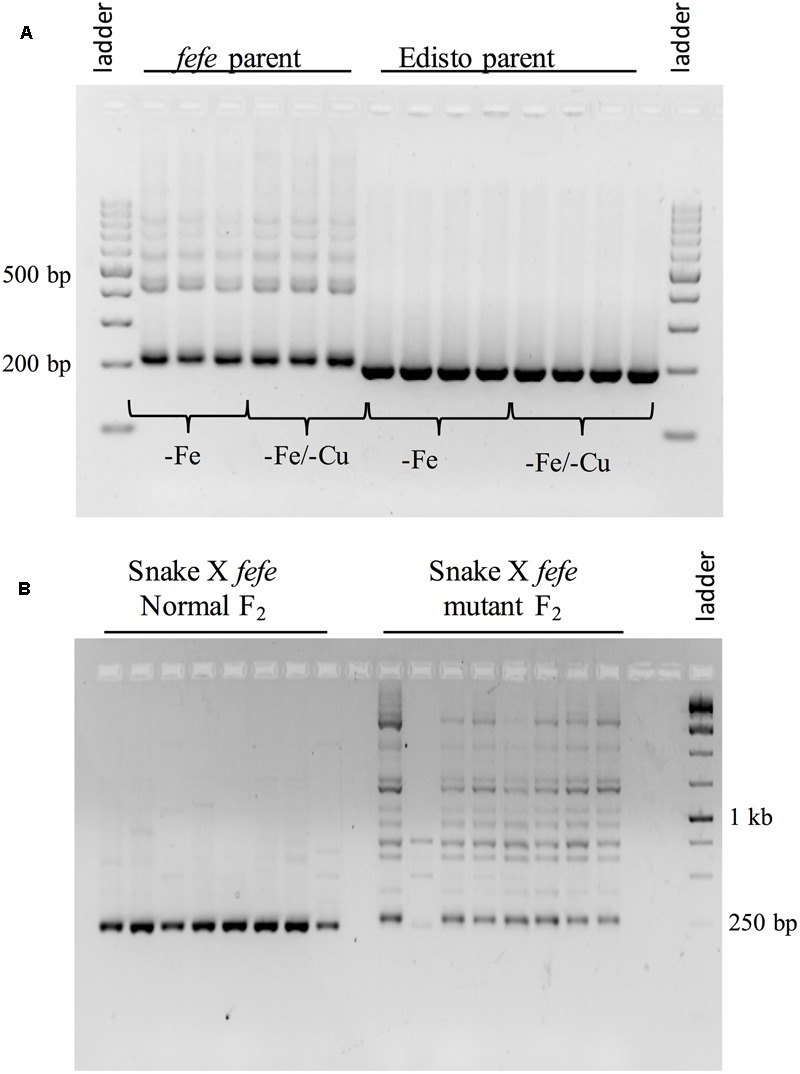
Reverse-transcriptase PCR of *bHLH38* using primers that span the retrotransposon insertion site. **(A)** Transcripts from roots of multiple individual plants of *fefe* or Edisto grown under Fe deficiency or simultaneous Fe and Cu deficiency; **(B)** Transcripts from roots of multiple individual plants of the snake melon X *fefe* F_2_ mapping population scored as normal leaf color (left) or chlorotic, indicating *fefe* mutant (right).

To better understand the expression of the *bHLH38* gene in *fefe* and WT plants, we visualized read mapping of *fefe* and Edisto *bHLH38* against the reference cucumber Cs*bHLH38* transcript (Csa4M434480.1). The reads mapped uniformly to the reference Cs*bHLH38* transcript in Edisto (**Figure 4A**), but the *fefe* read counts were much higher at the beginning of the transcript and decreased abruptly at position 370 bp of the reference transcript (**Figure 4B**), for both Fe deficient and Fe sufficient roots, suggesting the presence of a transcript variant. We performed *de novo* assembly of the Edisto and *fefe* transcriptomes to reconstruct the bHLH38 transcripts. The assembled WT *bHLH038* transcript was 943 bp, which includes predicted start and stop codons to include 309 deduced amino acids. The *de novo* assembly of the *fefe* mutant resulted in eight unique transcripts, which were longer than the WT transcript by 277–4789 bp. The extra length occurred beginning at 549 bp in the WT *bHLH038* transcript, where we saw read mapping anomalies relative to the cucumber transcript. Both before 549 bp and after the insertion, from 550 bp onward, the *fefe* transcript sequence matched the WT transcript sequence. Using RT-PCR with primers spanning the insertion site to visualize cDNA, a single band was present in Edisto, whereas the *fefe* parent contained multiple bands, in agreement with the *de novo* assembly results, in -Fe, and -Fe/-Cu treatments (**Figure 5A**). Similar to Fe deficiency, Cu deficiency did not change the *bHLH38* banding pattern. These results suggest that the *fefe* mutant was producing multiple insertion-containing *bHLH38* transcripts, or one large transcript that had been differently or partially spliced. A cDNA laddering pattern was also observed in the snake melon X *fefe* F_2_ mapping population (**Figure 5B**), but only in individual plants with the chlorotic *fefe* phenotype. The *fefe* cDNA band that was closest in size to the Edisto *bHLH38* cDNA was sequenced, to reveal the presence of a 14 bp insertion. Relative to the start codon, the *fefe* transcript had a reading frame shift followed by a premature in-frame stop codon, and would produce a different deduced amino acid sequence (Supplementary Data S2) that could negatively affect protein structure and function.

**FIGURE 6:**
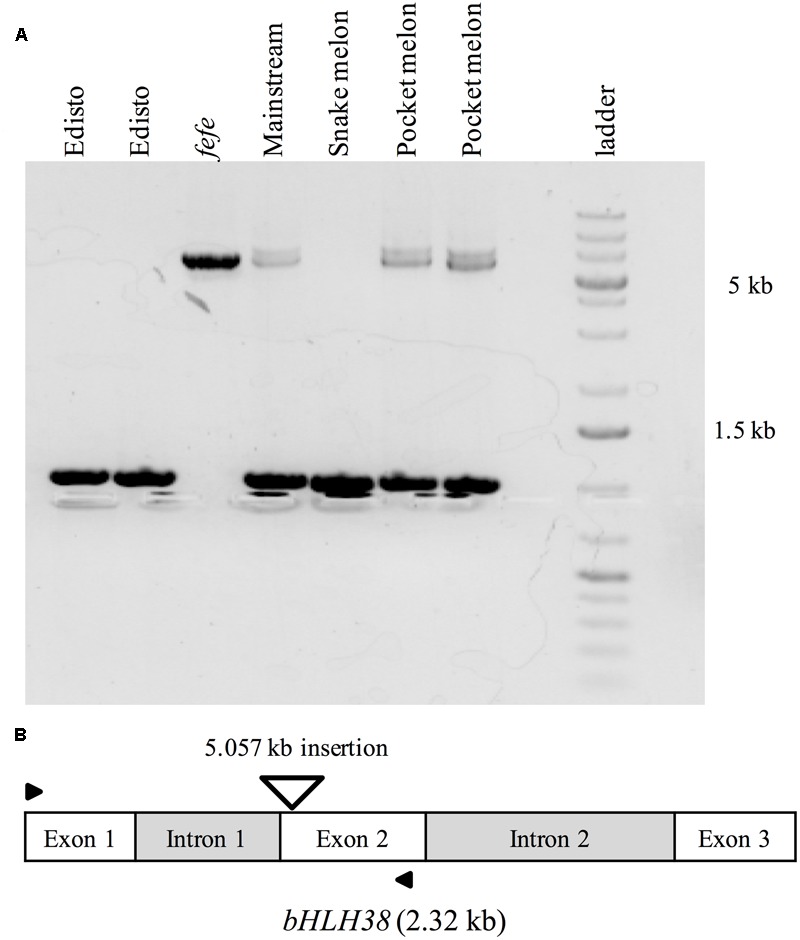
PCR amplification of genomic *bHLH38* from wild-type varieties and the *fefe* mutant. **(A)** From left to right, two individual plants of the wild-type Edisto, *fefe* mutant, wild-type Mainstream, wild-type snake melon, and two individual plants of the wild-type pocket melon; **(B)** Diagram depicting the melon *bHLH38* gene structure and insertion site of the 5.057 kb retrotransposon in *fefe bHLH38* (triangle). Arrows indicate the location of primers used for amplification of genomic *bHLH38* in **(A)**.

To further investigate the source of the transcript insertions in *fefe bHLH38*, we amplified genomic *bHLH38* in normal and *fefe* plants by PCR and sequenced the products. All WT lines produced an amplicon of 1.019 kb, while *fefe* produced a 6.076 kb fragment (**Figure 6**). The *fefe bHLH38* contained a 5.057 kb insertion relative to WT Edisto (Supplementary Data S3). The sequence of the insertion had identical 278 bp sequences at both extremes of the insertion (Supplementary Data S3 and Figure S1). A BLAST search of the *fefe* genomic *bHLH38* sequence against the melon reference genome had hits to six melon genomic scaffolds, unassembled sequences and scaffold 36. Length of the hits for *fefe bHLH38* using BLAST search against the melon genome ranged between 272 and 1368 bp, and the total scores from BLAST search ranged between 608 and 2510. An NCBI conserved domain search identified the insertion as a long terminal repeat (LTR) Ty1-copia type retrotransposon. The *fefe bHLH38* contained helix-loop-helix, polypurine tract, RNAseH1-RT-Ty1, Reverse transcriptase, Integrase, gag-polypeptide and primer binding site of LTR-copia type domains (Supplementary Figure S1). There were also large genomic bands in WTs Mainstream and pocket melon, of a similar but not identical size as the band in *fefe* (**Figure 6**). From the banding pattern and BLAST search, it appears that the retrotransposon in *fefe-bHLH38* could be present in other loci in the melon genome, potentially as an intact sequence. A global BLAST search for sequences similar to fefe *bHLH38* in the NCBI nucleotide database indicated that the *fefe bHLH38* retrotransposon was specific to melon.

### Complementation of the *fefe* Phenotype Using Hairy Root Transformation

**FIGURE 7:**
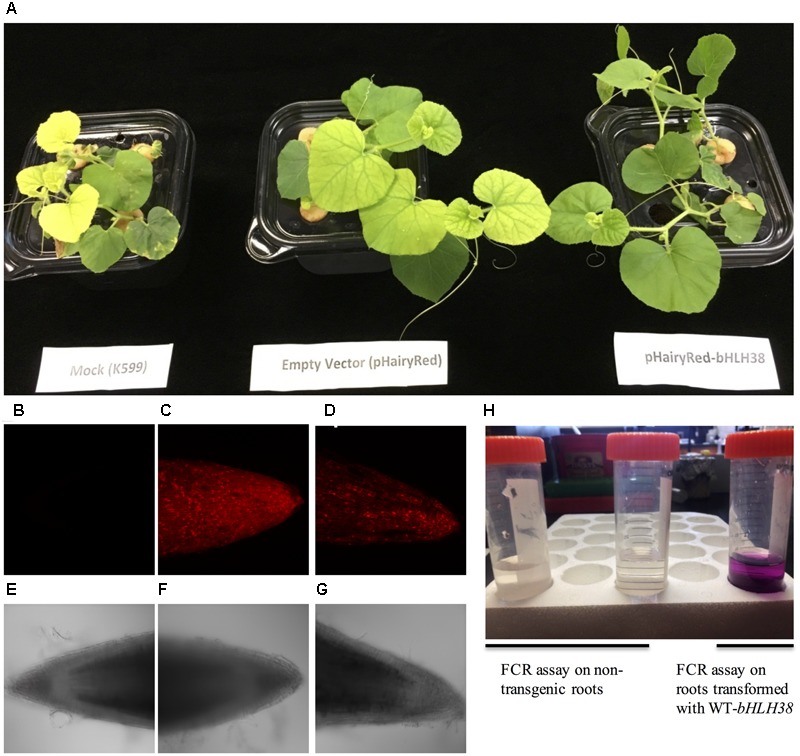
Complementation of the chlorotic *fefe* phenotype by hairy root transformation. **(A)** Plant mock-transformed with *Agrobacterium rhizogenes* K599, transformed with empty pHairyRed vector, and transformed with pHairyRed-Edisto-*bHLH038*. **(B–D)** *DsRed* expression in hairy roots of *fefe* plants viewed by confocal microscopy, **(B)** mock transformation, **(C)** pHairyRed and **(D)** pHairyRed-Edisto-*bHLH038*. Bright field view of same roots in **(B–D)**; **(E)** mock transformation, **(F)** pHairyRed and **(G)** pHairyRed-Edisto-*bHLH038* roots; **(H)** Ferric chelate reductase activity of rescued *fefe* plant roots transformed with pHairyRed-Edisto-*bHLH38* compared to non-transgenic roots.

Since the *FeFe* gene is necessary for normal plant growth and Fe uptake only in roots (Waters et al., 2014), we tested whether the chlorotic *fefe* phenotype could be complemented with a normal copy of *bHLH38*. We transformed *fefe* plants with *Agrobacterium rhizogenes* to generate hairy roots containing the WT *bHLH38* gene (**Figure 7**). The chlorotic phenotype of the *fefe* plants was rescued in the pHairyRed-Edisto-*bHLH38* treated plants, but not in mock or empty vector treated plants (**Figure 7A**). The rescued *fefe* plant roots were positive for the dsRed reporter gene (**Figures 7B–G**), and were able to initiate ferric reductase activity under Fe deficiency (**Figure 7H**), suggesting that normal root Fe uptake responses were recovered in the transgenic *fefe* plant roots expressing Edisto-*bHLH38.*

### Yeast Two-hybrid Assays for Protein–Protein Interactions

**FIGURE 8:**
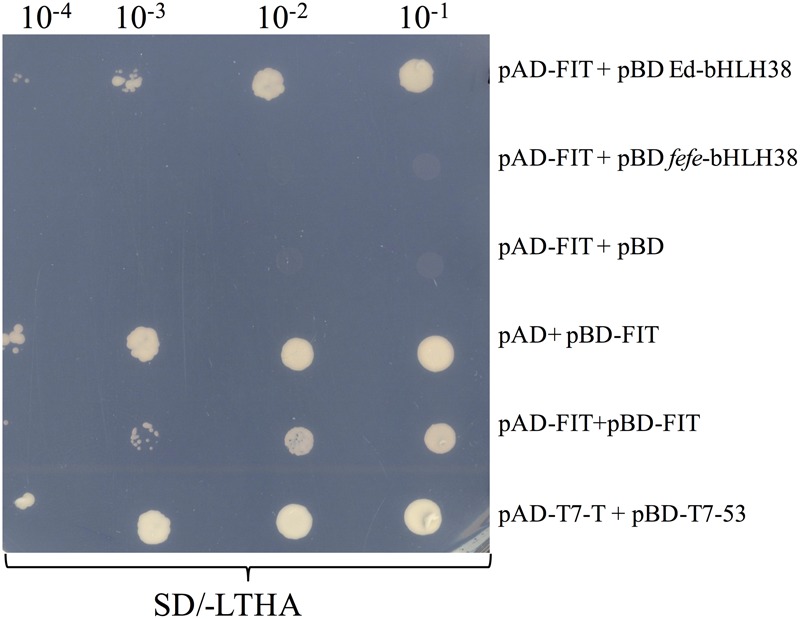
Yeast 2-hybrid assay of bHLH38 and Fit in Edisto (ed) and *fefe* plated on SD media without Leu, Trp, His, and Ade in a serial dilution series. pAD-FIT+pBD ed-bHLH38 tests the interaction of FIT with Edisto-bHLH38, pAD-FIT+pBD fefe-bHLH38 tests the interaction of FIT with fefe-bHLH38, pAD-FIT+pBD and pAD+pBD-FIT are controls to test if FIT is capable of auto-activation, pAD-FIT+pBD-FIT is a test to check homodimer formation of FIT. pAD-T7-T+pBD-T7-53 is a positive control for the assay.

To determine whether the bHLH38 protein interacts with the Fit protein, as it does in Arabidopsis (Yuan et al., 2008), we performed a yeast two-hybrid experiment. Yeast cells transformed with different combinations of bait and prey plasmids were tested for auxotrophic growth. The melon Fit protein tested positive for interacting with Edisto bHLH38, but did not interact with *fefe* bHLH38 (**Figure 8**). The Edisto bHLH38 protein was capable of forming a homodimer, although yeast growth was less robust than in other combinations (Supplementary Figure S2A). The *fefe* bHLH38 did not form homodimers (Supplementary Figure S2B). Yeast growth suggested that there was some degree of interaction between Edisto bHLH38 and *fefe* bHLH38. This test could not be used to determine whether melon Fit forms a homodimer, since Fit was capable of auto-activation, as indicated by growth of yeast transformed with pAD+pBD-FIT (**Figure 8**).

## Discussion

In this paper, we seek new understanding of how Strategy I plants respond to Fe-deficiency stress by mapping the *fefe* gene that controls Fe uptake in melon. We mapped the *fefe* gene to *bHLH38*, which contains a 5.056 kb Ty1-copia type retrotransposon insertion. Multiple length transcripts were observed in *fefe-bHLH38* (**Figures 5A,B**), apparently due to the full retrotransposon being incorporated into the transcript and partially spliced out to varying degrees. The altered RNA-seq read mapping we observed, with about three times higher reads before the retrotransposon insertion site in *fefe-bHLH38* (**Figures 3, 4**), may arise from promoters within LTR regions of LTR retrotransposons (Kumar and Bennetzen, 1999). Coincidentally, the loss of Fe homeostasis in the tomato *fer* mutant (Ling et al., 2002) is due to an insertion of a copia-type retrotransposon, called *Rider*, in the first exon of the *FER* gene (Cheng et al., 2009). *Rider* replicates by reverse transcribing an aberrant and novel cDNA that can include nearby genes, and this novel cDNA is then integrated into a new location in the genome (Lisch, 2012). We saw evidence for a pseudogene in some varieties of melon (Mainstream and pocket melon) that was amplified by PCR primers located in the exons of the *bHLH38* gene (**Figure 6**), suggesting that the retrotransposon in *fefe* exists in other loci in the melon genome. To our knowledge this retrotransposon has not been described in detail, since BLAST searches of the reference melon genome only hit unassembled scaffolds, however, it could be an important feature in melon evolution and diversity (Kumar and Bennetzen, 1999).

Regulation of Fe-uptake genes, such as *FRO1, Nramp1*, and *IRT1*, was abolished in the *fefe* mutant (Waters et al., 2014), similar to the *Fit* mutant of Arabidopsis (Colangelo and Guerinot, 2004) and the *fer* mutant of tomato (Ling et al., 2002), however, the lesion in the *fefe* mutant is not in the *FIT* gene (Waters et al., 2014). The formation of a heterodimer between the Fit protein and subgroup Ib bHLH proteins is a hallmark of transcriptional regulation of Fe-uptake genes (Yuan et al., 2008; Du et al., 2015). The *fefe-bHLH38* transcript that was closest in size to the normal *bHLH38* transcript would be translated to a protein that has an altered sequence after the first 120 aa, and terminates at 144 aa instead of 249 aa, due to the 14-bp insertion (Supplementary Data S2). Using the yeast 2-hybrid technique, the WT-bHLH38 interacted with the Fit protein, however, the fefe-bHLH38 protein did not form a heterodimer with the Fit protein (**Figure 8**). We propose that this lack of Fit-bHLH38 heterodimer formation is the cause of abolished upregulation of Fe-uptake gene expression in *fefe*. The severity of the *fefe* phenotype under Fe sufficient conditions from mutation of a single *bHLH* gene is surprising considering that in Arabidopsis, single, double, or even triple *bHLH* mutants (with *bHLH38* or *bHLH39* remaining and the other three subgroup Ib *bHLH* genes knocked out) had no Fe deficiency phenotype under Fe sufficient conditions (Wang et al., 2007; Sivitz et al., 2012; Andriankaja et al., 2014; Maurer et al., 2014), suggesting that one of the four bHLH proteins is adequate for Fe-uptake gene regulation. The tomato genome has three subgroup Ib *bHLH* genes (Sun et al., 2015), and the soybean genome has two subgroup Ib *bHLH* genes. A 12 bp deletion in one of the soybean *bHLH* genes was suggested to cause increased sensitivity to alkalinity-induced Fe deficiency chlorosis (Peiffer et al., 2012). But, like the Arabidopsis *bHLH* mutants, the soybean lines with this deletion are not chlorotic under Fe sufficient conditions (O’Rourke et al., 2007). A knockout line for the soybean *bHLH* genes has not been reported, and quadruple Arabidopsis *bHLH* lines have not been generated. So far, *fefe* is the only subgroup Ib *bHLH* mutant with an Fe uptake phenotype as severe as the *fit* or *fer* mutants. While the melon genome has a second subgroup Ib *bHLH* gene, *bHLH101* was not polymorphic between WT and the *fefe* mutant, its transcript abundance was strikingly low, which together with genetic results suggests that melon *bHLH101* is not functionally redundant with melon *bHLH38*.

Another key difference between melon *bHLH38* and subgroup Ib *bHLH* genes in other plant species is their transcriptional regulation in roots by Fe status. In Arabidopsis and tomato, subgroup Ib *bHLH* genes are upregulated upon Fe deficiency in roots (Wang et al., 2007; Dinneny et al., 2008; Buckhout et al., 2009; Yang et al., 2010; Bauer and Blondet, 2011; Schuler et al., 2011; Stein and Waters, 2012; Waters et al., 2012; Andriankaja et al., 2014; Maurer et al., 2014; Sun et al., 2015). However, *bHLH38* was not upregulated in Fe deficient melon roots (**Figure 3**; Waters et al., 2014; Hsieh and Waters, 2016). Copper deficiency also did not change melon *bHLH38* expression, or its transcript pattern in *fefe* (**Figure 5A**), suggesting that the rescue of the chlorotic phenotype of *fefe* under simultaneous Fe and Cu deficiency (Waters et al., 2014) was not due to a change in *bHLH38* expression, splicing, or protein function. However, since the bHLH38 protein is crucial to Fe homeostasis, its regulation may be entirely at the post-transcriptional or post-translational level. Arabidopsis *FIT* is regulated at both the transcriptional and post-transcriptional levels (Meiser et al., 2011; Sivitz et al., 2011).

We confirmed that the *fefe* defect in root Fe-uptake is due to loss of function of *bHLH38* by complementation of the *fefe* chlorotic phenotype with WT-*bHLH38* (**Figure 7**). The mapping and identification of the *fefe* mutation as *bHLH38* has given new insight into regulation of Fe homeostasis in Strategy I plants. The *fefe* mutant may prove to be a valuable platform for studying *bHLH* genes and proteins from other plant species, since it can be complemented by hairy root transformation. Further characterization of bHLH38 protein regulation in melon is a needed future direction to help provide understanding of Fe-uptake control mechanisms.

## Author Contributions

RR and BW planned and designed experiments. RR conducted experiments and performed statistical and bioinformatics analysis. RR and BW wrote the manuscript. All authors read and approved the whole manuscript.

## Conflict of Interest Statement

The authors declare that the research was conducted in the absence of any commercial or financial relationships that could be construed as a potential conflict of interest.

## Footnotes

1 http://www.icugi.org/cgi-bin/cmap/map_set_info?species_acc=CM

2 https://trace.ncbi.nlm.nih.gov/Traces/study/?acc=PRJNA371826&go=go

3 http://www.broadinstitute.org/igv

## References

[B1] Anders S. Huber W. (2010). Differential expression analysis for sequence count data. *Genome Biol.* 11:R106 10.1186/gb-2010-11-10-r106 PMC3218662 20979621

[B2] Andriankaja M. E. Danisman S. Mignolet-Spruyt L. F. Claeys H. Kochanke I. Vermeersch M. (2014). Transcriptional coordination between leaf cell differentiation and chloroplast development established by TCP20 and the subgroup Ib bHLH transcription factors. *Plant Mol. Biol.* 85 233–245. 10.1007/s11103-014-0180-2 24549883

[B3] Bashir K. Nishizawa N. K. (2013). “Iron proteins, plant iron transporters,” in *Encyclopedia of Metalloproteins* eds Kretsinger R. H. Uversky V. N. Permyakov E. A. (New York, NY: Springer) 1015–1023. 10.1007/978-1-4614-1533-6_356

[B4] Bauer P. Blondet E. (2011). Transcriptome analysis of *ein3 eil1* mutants in response to iron deficiency. *Plant Signal. Behav.* 6 1669–1671. 10.4161/psb.6.11.17847 22212120 PMC3329332

[B5] Benjamini Y. Hochberg Y. (1995). Controlling the false discovery rate: a practical and powerful approach to multiple testing. *J. R. Stat. Soc. Ser. B* 57 289–300. 10.2307/2346101

[B6] Briat J. F. Dubos C. Gaymard F. (2015). Iron nutrition, biomass production, and plant product quality. *Trends Plant Sci.* 20 33–40. 10.1016/j.tplants.2014.07.005 25153038

[B7] Broman K. W. Wu H. Sen S. Churchill G. A. (2003). R/qtl: QTL mapping in experimental crosses. *Bioinformatics* 19 889–890. 12724300 10.1093/bioinformatics/btg112

[B8] Buckhout T. J. Yang T. J. Schmidt W. (2009). Early iron-deficiency-induced transcriptional changes in *Arabidopsis* roots as revealed by microarray analyses. *BMC Genomics* 10:147 10.1186/1471-2164-10-147 PMC2676303 19348669

[B9] Castaings L. Caquot A. Loubet S. Curie C. (2016). The high-affinity metal transporters NRAMP1 and IRT1 team up to take up iron under sufficient metal provision. *Sci. Rep.* 6:37222 10.1038/srep37222 PMC5110964 27849020

[B10] Chabaud M. Boisson-Dernier A. Zhang J. Taylor C. G. Yu O. Barker D. G. (2006). “Agrobacterium rhizogenes-mediated root transformation,” in *The Medicago Truncatula Handbook, Version November* eds Mathesius U. Journer E. P. Sumne L. W. (Ardmore, OK: The Samuel Roberts Noble Foundation).

[B11] Chen Y. Barak P. (1982). Iron nutrition of plants in calcareous soils. *Adv. Agron.* 35 217–240.

[B12] Cheng X. Zhang D. Cheng Z. Keller B. Ling H.-Q. (2009). A new family of Ty1-copia-like retrotransposons originated in the tomato genome by a recent horizontal transfer event. *Genetics* 181 1183–1193. 10.1534/genetics.108.099150 19153256 PMC2666490

[B13] Colangelo E. P. Guerinot M. L. (2004). The essential basic helix-loop-helix protein FIT1 is required for the iron deficiency response. *Plant Cell* 16 3400–3412. 10.1105/tpc.104.024315 15539473 PMC535881

[B14] Curie C. Alonso J. M. Le Jean M. Ecker J. R. Briat J. F. (2000). Involvement of NRAMP1 from *Arabidopsis thaliana* in iron transport. *Biochem. J.* 347 749–755. 10769179 PMC1221012

[B15] Curie C. Panaviene Z. Loulergue C. Dellaporta S. L. Briat J. F. Walker E. L. (2001). Maize yellow stripe1 encodes a membrane protein directly involved in Fe(III) uptake. *Nature* 409 346–349. 10.1038/35053080 11201743

[B16] Diaz A. Fergany M. Formisano G. Ziarsolo P. Blanca J. Fei Z. (2011). A consensus linkage map for molecular markers and quantitative trait loci associated with economically important traits in melon (*Cucumis melo* L.). *BMC Plant Biol.* 11:111 10.1186/1471-2229-11-111 PMC3163537 21797998

[B17] Dinneny J. R. Long T. A. Wang J. Y. Jung J. W. Mace D. Pointer S. (2008). Cell identity mediates the response of *Arabidopsis* roots to abiotic stress. *Science* 320 942–945. 10.1126/science.1153795 18436742

[B18] Du J. Huang Z. Wang B. Sun H. Chen C. Ling H.-Q. (2015). SlbHLH068 interacts with FER to regulate the iron-deficiency response in tomato. *Ann. Bot.* 116 23–34. 10.1093/aob/mcv058 26070639 PMC4479748

[B19] Garcia-Mas J. Benjak A. Sanseverino W. Bourgeois M. Mir G. González V. M. (2012). The genome of melon (*Cucumis melo* L.). *Proc. Natl. Acad. Sci. U.S.A.* 109 11872–11877. 10.1073/pnas.1205415109 22753475 PMC3406823

[B20] González V. M. Rodríguez-Moreno L. Centeno E. Benjak A. Garcia-Mas J. Puigdomènech P. (2010). Genome-wide BAC-end sequencing of *Cucumis melo* using two BAC libraries. *BMC Genomics* 11:618 10.1186/1471-2164-11-618 PMC3091759 21054843

[B21] Haas B. J. Papanicolaou A. Yassour M. Grabherr M. Blood P. D. Bowden J. (2013). De novo transcript sequence reconstruction from RNA-seq using the Trinity platform for reference generation and analysis. *Nat. Protoc.* 8 1494–1512. 10.1038/nprot.2013.084 23845962 PMC3875132

[B22] Hsieh E.-J. Waters B. M. (2016). Alkaline stress and iron deficiency regulate iron uptake and riboflavin synthesis gene expression differently in root and leaf tissue: implications for iron deficiency chlorosis. *J. Exp. Bot.* 67 5671–5685. 27605716 10.1093/jxb/erw328 PMC5066488

[B23] Huang S. Li R. Zhang Z. Li L. Gu X. Fan W. (2009). The genome of the cucumber, *Cucumis sativus* L. *Nat. Genet.* 41 1275–1281. 10.1038/ng.475 19881527

[B24] Kang H. Cho Y. Yoon U. Eun M. (1998). A rapid DNA extraction method for RFLP and PCR analysis from a single dry seed. *Plant Mol. Biol. Rep.* 16 1–9. 10.1023/a:1007418606098

[B25] Kereszt A. Li D. Indrasumunar A. Nguyen C. D. Nontachaiyapoom S. Kinkema M. (2007). Agrobacterium rhizogenes-mediated transformation of soybean to study root biology. *Nat. Protoc.* 2 948–952. 10.1038/nprot.2007.141 17446894

[B26] Kobayashi T. Nishizawa N. K. (2012). Iron uptake, translocation, and regulation in higher plants. *Annu. Rev. Plant Biol.* 63 131–152. 10.1146/annurev-arplant-042811-105522 22404471

[B27] Kumar A. Bennetzen J. L. (1999). Plant retrotransposons. *Annu. Rev. Genet.* 33 479–532. 10.1146/annurev.genet.33.1.479 10690416

[B28] Langmead B. Salzberg S. L. (2012). Fast gapped-read alignment with Bowtie 2. *Nat. Methods* 9 357–359. 10.1038/nmeth.1923 22388286 PMC3322381

[B29] Li D. Cuevas H. E. Yang L. Li Y. Garcia-Mas J. Zalapa J. (2011). Syntenic relationships between cucumber (*Cucumis sativus* L.) and melon (*C. melo* L.) chromosomes as revealed by comparative genetic mapping. *BMC Genomics* 12:396 10.1186/1471-2164-12-396 PMC3199783 21816110

[B30] Li H. Handsaker B. Wysoker A. Fennell T. Ruan J. Homer N. (2009). The sequence alignment/map format and SAMtools. *Bioinformatics* 25 2078–2079. 10.1093/bioinformatics/btp352 19505943 PMC2723002

[B31] Lin M.-H. Gresshoff P. M. Indrasumunar A. Ferguson B. J. (2011). pHairyRed: a novel binary vector containing the DsRed2 reporter gene for visual selection of transgenic hairy roots. *Mol. Plant* 4 537–545. 10.1093/mp/ssq084 21324970

[B32] Ling H.-Q. Bauer P. Bereczky Z. Keller B. Ganal M. (2002). The tomato fer gene encoding a bHLH protein controls iron-uptake responses in roots. *Proc. Natl. Acad. Sci. U.S.A.* 99 13938–13943. 10.1073/pnas.212448699 12370409 PMC129801

[B33] Lisch D. (2012). How important are transposons for plant evolution? *Nat. Rev. Genet.* 14 49–61. 10.1038/nrg3374 23247435

[B34] Marschner H. Romheld V. Kissel M. (1986). Different strategies in higher plants in mobilization and uptake of iron. *J. Plant Nutr.* 9 695–713. 10.1080/01904168609363475

[B35] Maurer F. Naranjo Arcos M. A. Bauer P. (2014). Responses of a triple mutant defective in three iron deficiency-induced Basic Helix-Loop-Helix genes of the subgroup Ib(2) to iron deficiency and salicylic acid. *PLoS ONE* 9:e99234 10.1371/journal.pone.0099234 PMC4053374 24919188

[B36] Meiser J. Lingam S. Bauer P. (2011). Posttranslational regulation of the iron deficiency basic helix-loop-helix transcription factor FIT is affected by iron and nitric oxide. *Plant Physiol.* 157 2154–2166. 10.1104/pp.111.183285 21972265 PMC3327203

[B37] Nugent P. E. (1994). Iron chlorotic melon germplasm C940-fe. *HortScience* 29 50–51.

[B38] Nugent P. E. Bhella H. S. (1988). A new chlorotic mutant of muskmelon. *HortScience* 23 379–381.

[B39] O’Rourke J. A. Charlson D. V. Gonzalez D. O. Vodkin L. O. Graham M. A. Cianzio S. R. (2007). Microarray analysis of iron deficiency chlorosis in near-isogenic soybean lines. *BMC Genomics* 8:476 10.1186/1471-2164-8-476 PMC2253546 18154662

[B40] Peiffer G. A. King K. E. Severin A. J. May G. D. Cianzio S. R. Lin S. F. (2012). Identification of candidate genes underlying an iron efficiency quantitative trait locus in soybean. *Plant Physiol.* 158 1745–1754. 10.1104/pp.111.189860 22319075 PMC3320182

[B41] Pillet J. Yu H.-W. Chambers A. H. Whitaker V. M. Folta K. M. (2015). Identification of candidate flavonoid pathway genes using transcriptome correlation network analysis in ripe strawberry (*Fragaria* × *ananassa*) fruits. *J. Exp. Bot.* 66 4455–4467. 10.1093/jxb/erv205 25979996 PMC4507756

[B42] Ramamurthy R. K. Waters B. M. (2015). Identification of fruit quality and morphology QTLs in melon (*Cucumis melo*) using a population derived from flexuosus and cantalupensis botanical groups. *Euphytica* 204 163–177. 10.1007/s10681-015-1361-z

[B43] Robinson N. J. Procter C. M. Connolly E. L. Guerinot M. L. (1999). A ferric-chelate reductase for iron uptake from soils. *Nature* 397 694–697. 10067892 10.1038/17800

[B44] Römheld V. Marschner H. (1986). Evidence for a specific uptake system for iron phytosiderophores in roots of grasses. *Plant Physiol.* 80 175–180. 16664577 10.1104/pp.80.1.175 PMC1075078

[B45] Santi S. Schmidt W. (2009). Dissecting iron deficiency-induced proton extrusion in *Arabidopsis* roots. *New Phytol.* 183 1072–1084. 10.1111/j.1469-8137.2009.02908.x 19549134

[B46] Schuler M. Keller A. Backes C. Philippar K. Lenhof H.-P. Bauer P. (2011). Transcriptome analysis by GeneTrail revealed regulation of functional categories in response to alterations of iron homeostasis in *Arabidopsis thaliana*. *BMC Plant Biol.* 11:87 10.1186/1471-2229-11-87 PMC3114716 21592396

[B47] Seo P. J. Lee S. B. Suh M. C. Park M.-J. Go Y. S. Park C.-M. (2011). The MYB96 transcription factor regulates cuticular wax biosynthesis under drought conditions in *Arabidopsis*. *Plant Cell* 23 1138–1152. 10.1105/tpc.111.083485 21398568 PMC3082259

[B48] Sivitz A. Grinvalds C. Barberon M. Curie C. Vert G. (2011). Proteasome-mediated turnover of the transcriptional activator FIT is required for plant iron-deficiency responses. *Plant J.* 66 1044–1052. 10.1111/j.1365-313X.2011.04565.x 21426424

[B49] Sivitz A. B. Hermand V. Curie C. Vert G. (2012). *Arabidopsis* bHLH100 and bHLH101 control iron homeostasis via a FIT-independent pathway. *PLoS ONE* 7:e44843 10.1371/journal.pone.0044843 PMC3439455 22984573

[B50] Stein R. J. Waters B. M. (2012). Use of natural variation reveals core genes in the transcriptome of iron-deficient *Arabidopsis thaliana* roots. *J. Exp. Bot.* 63 1039–1055. 10.1093/jxb/err343 22039296 PMC3254695

[B51] Sun H. Fan H.-J. Ling H.-Q. (2015). Genome-wide identification and characterization of the bHLH gene family in tomato. *BMC Genomics* 16:9 10.1186/s12864-014-1209-2 PMC4312455 25612924

[B52] To J. P. C. Haberer G. Ferreira F. J. Deruère J. Mason M. G. Schaller G. E. (2004). Type-A *Arabidopsis* response regulators are partially redundant negative regulators of cytokinin signaling. *Plant Cell* 16 658–671. 10.1105/tpc.018978 14973166 PMC385279

[B53] Varotto C. Maiwald D. Pesaresi P. Jahns P. Salamini F. Leister D. (2002). The metal ion transporter IRT1 is necessary for iron homeostasis and efficient photosynthesis in *Arabidopsis thaliana*. *Plant J.* 31 589–599. 12207649 10.1046/j.1365-313x.2002.01381.x

[B54] Verelst W. Twell D. de Folter S. Immink R. Saedler H. Münster T. (2007). MADS-complexes regulate transcriptome dynamics during pollen maturation. *Genome Biol.* 8:R249 10.1186/gb-2007-8-11-r249 PMC2258202 18034896

[B55] Vert G. Grotz N. Dédaldéchamp F. Gaymard F. Guerinot M. L. Briat J.-F. (2002). IRT1, an *Arabidopsis* transporter essential for iron uptake from the soil and for plant growth. *Plant Cell* 14 1223–1233. 10.1105/TPC.001388 12084823 PMC150776

[B56] Von Jolley D. Brown J. C. Nugent P. E. (1991). A genetically related response to iron deficiency stress in muskmelon. *Plant Soil* 130 87–92. 10.1007/BF00011860

[B57] Wang H.-Y. Klatte M. Jakoby M. Bäumlein H. Weisshaar B. Bauer P. (2007). Iron deficiency-mediated stress regulation of four subgroup Ib BHLH genes in *Arabidopsis thaliana*. *Planta* 226 897–908. 10.1007/s00425-007-0535-x 17516080

[B58] Wang N. Cui Y. Liu Y. Fan H. Du J. Huang Z. (2013). Requirement and functional redundancy of Ib subgroup bHLH proteins for iron deficiency responses and uptake in *Arabidopsis thaliana*. *Mol. Plant* 6 503–513. 10.1093/mp/sss089 22983953

[B59] Waters B. M. Lucena C. Romera F. J. Jester G. G. Wynn A. N. Rojas C. L. (2007). Ethylene involvement in the regulation of the H+-ATPase CsHA1 gene and of the new isolated ferric reductase CsFRO1 and iron transporter CsIRT1 genes in cucumber plants. *Plant Physiol. Biochem.* 45 293–301. 10.1016/j.plaphy.2007.03.011 17468001

[B60] Waters B. M. McInturf S. A. Amundsen K. (2014). Transcriptomic and physiological characterization of the fefe mutant of melon (*Cucumis melo*) reveals new aspects of iron-copper crosstalk. *New Phytol.* 203 1128–1145. 10.1111/nph.12911 24975482 PMC4117724

[B61] Waters B. M. McInturf S. A. Stein R. J. (2012). Rosette iron deficiency transcript and microRNA profiling reveals links between copper and iron homeostasis in *Arabidopsis thaliana*. *J. Exp. Bot.* 63 5903–5918. 10.1093/jxb/ers239 22962679 PMC3467300

[B62] Welkie G. W. (1996). Iron-deficiency stress responses of a chlorosis-susceptible and a chlorosis-resistant cultivar of muskmelon as related to root riboflavin excretion. *J. Plant Nutr.* 19 1157–1169. 10.1080/01904169609365187

[B63] Wise A. A. Liu Z. Binns A. N. (2006). “Three methods for the introduction of foreign DNA into *Agrobacterium*,” in *Agrobacterium Protocols* ed. Wang K. (New York, NY: Humana Press) 43–54. 10.1385/1-59745-130-4:43 16988332

[B64] Yang T. J. W. Lin W.-D. Schmidt W. (2010). Transcriptional profiling of the *Arabidopsis* iron deficiency response reveals conserved transition metal homeostasis networks. *Plant Physiol.* 152 2130–2141. 10.1104/pp.109.152728 20181752 PMC2850031

[B65] Yuan Y. Wu H. Wang N. Li J. Zhao W. Du J. (2008). FIT interacts with AtbHLH38 and AtbHLH39 in regulating iron uptake gene expression for iron homeostasis in *Arabidopsis*. *Cell Res.* 18 385–397. 10.1038/cr.2008.26 18268542

[B66] Yuan Y. X. Zhang J. Wang D. W. Ling H. Q. (2005). AtbHLH29 of *Arabidopsis thaliana* is a functional ortholog of tomato FER involved in controlling iron acquisition in strategy I plants. *Cell Res.* 15 613–621. 10.1038/sj.cr.7290331 16117851

