# Supplementary material for: Mapping and Characterization of the fefe Gene That Controls Iron Uptake in Melon (Cucumis melo L.)
Source: Front Plant Sci. 2017 Jun 14;8:1003. doi: 10.3389/fpls.2017.01003 (PMC5470102; doi:10.3389/fpls.2017.01003)
Supplement: Supplementary file 2 [file Data_Sheet_2.docx]

Supplementary Material

**Mapping and characterization of the *fefe* gene that controls iron uptake in melon (*Cucumis melo*. L)**

Raghuprakash Kastoori Ramamurthy and Brian M. Waters

*** Correspondence:** Corresponding Author: Brian M. Waters (bwaters2@unl.edu)

10 20 30 40 50

....|....| ....|....| ....|....| ....|....| ....|....|

fefe.bHLH38 ---------- -------MLA VSSPLFSPHQ WQLEDPISLH HQHNSLFSPF

Edisto.bHLH38 ---------- -------MLA VSSPLFSPHQ WQLEDPISLH HQHNSLFSPF

AtbHLH038 ---------- -------MCA LVPSFFTNFG WPSTN----- -QYESYYGAG

AtbHLH039 ---------- -------MCA LVPPLFPNFG WPSTG----- -EYDSYYLAG

AtbHLH100 ---------- -------MCA LVPPLYPNFG WPCG------ --DHSFYETD

AtbHLH101 ---------- ---------M EYPWLQSQVH SFSPTLHFPS FLHPLDD---

Cm-bHLH101 ---------- ---------- ---------- ---------- ----------

SlbHLH068 MLAFSSSNMF PTMNSIAWSL EEPLSYDDHH KNTTIITTPQ FQTDQNNKLF

60 70 80 90 100

....|....| ....|....| ....|....| ....|....| ....|....|

fefe.bHLH38 EPSDHSFYLQ FPPP------ ---PLDPSHD HYPSSAAPSP EAVSNVSKMA

Edisto.bHLH38 EPSDHSFYLQ FPPP------ ---PLDPSHD HYPSSAAPSP EAVSNVSKMA

AtbHLH038 DNLNNGTFLE LTVP-QTY-- --EVTHHQNS LGVSVSSEGN -EIDNNPVVV

AtbHLH039 DILNNGGFLD FPVPEETYGA VTAVTQHQNS FGVSVSSEGN -EIDNNPVVV

AtbHLH100 DVSN--TFLD FPLP------ -DLTVTHEN- ----VSSENN RTLLDNPVVM

AtbHLH101 ---SKSHNIN LHHM------ ----SLSHSN NTNSNNNNYQ EEDRGAVVLE

Cm-bHLH101 ---------- ---------- ---------- ---------- ----------

SlbHLH068 EGLRADNTID LPSS------ ----HHYQQQ CLKGSEFDVD ELGVERSLME

110 120 130 140 150

....|....| ....|....| ....|....| ....|....| ....|....|

fefe.bHLH38 KKLSHNASER DRRKKINSLY SSLRALLP-- -SSDQMKKLS NPATISRILL

Edisto.bHLH38 KKLSHNASER DRRKKINSLY SSLRALLP-- -SSDQMKKLS NPATISRILS

AtbHLH038 KKLNHNASER DRRKKINTLF SSLRSCLP-- -ASDQSKKLS IPETVSKSLK

AtbHLH039 KKLNHNASER DRRRKINSLF SSLRSCLP-- -ASGQSKKLS IPATVSRSLK

AtbHLH100 KKLNHNASER ERRKKINTMF SSLRSCLP-- -PTNQTKKLS VSATVSQALK

AtbHLH101 KKLNHNASER DRRRKLNALY SSLRALLP-- -LSDQKRKLS IPMTVARVVK

Cm-bHLH101 ---------- ---------- ---------- --------MS NPSTISKALK

SlbHLH068 KKLNHNASER NRRKKMNFLY STLRSLLPPP TNKHQKKKLS FPATVSYVQE

160 170 180 190 200

....|....| ....|....| ....|....| ....|....| ....|....|

fefe.bHLH38 KIFMVSEQFD PNFK------ ---------- ---------- ----------

Edisto.bHLH38 YIPELQQQVE GQMRKKEELM AAMVG----Q EVKNDEEKKM KSAASSSSSI

AtbHLH038 YIPELQQQVK RLIQKKEEIL VRVSG----Q RDFELYDK-- QQPKAVASYL

AtbHLH039 YIPELQEQVK KLIKKKEELL VQISG----Q RNTECYVK-- QPPKAVANYI

AtbHLH100 YIPELQEQVK KLMKKKEELS FQISG----Q RDLVYTDQNS KSEEGVTSYA

AtbHLH101 YIPEQKQELQ RLSRRKEELL KRISR----K THQEQLRNKA MMDSIDSSSS

Cm-bHLH101 YIPELQQQVE GLRRRKEGLV TKLN-----E ENLKQIR--- -KNNKEPWMS

SlbHLH068 YIPELKKEIE RLSKTKDLLL SKKSNYSLLK IDDNNKRKLI IGGTSCNSST

210 220 230 240 250

....|....| ....|....| ....|....| ....|....| ....|....|

fefe.bHLH38 ---------- ----LSDQAQ RCH------- ---------- ----------

Edisto.bHLH38 ISASRLSRHE MAIQISTDIN GCQR--NYLS EILCCLEEEG LLLLNASSFE

AtbHLH038 STVSATRLGD NEVMVQVSSS KIHN--FSIS NVLGGIEEDG FVLVDVSSSR

AtbHLH039 STVSATRLGD NEVMVQISSS KIHN--FSIS NVLSGLEEDR FVLVDMSSSR

AtbHLH100 STVSSTRLSE TEVMVQISSL QTEK--CSFG NVLSGVEEDG LVLVGASSSR

AtbHLH101 QRIAANWLTD TEIAVQIATS KWTS----VS DMLLRLEENG LNVISVSSSV

Cm-bHLH101 SLCAVNWLSE TEALLQIALE EQTHTQLPFS QILLSLEDDG LLLSTASSFR

SlbHLH068 TSICASQLSN SQVLVQISTT QENN--FPIS QVFASVEEDG LILLNASSFK

260 270 280 290

....|....| ....|....| ....|....| ....|....| ....|

fefe.bHLH38 ---------- ---------- ---------- ---------- -----

Edisto.bHLH38 SFDGKVFHNL HLQMG--SNC KMEPKVLSNM LLEMFP---- -----

AtbHLH038 SQGERLFYTL HLQVENMDDY KINCEELSER MLYLYEKCEN SFN--

AtbHLH039 SQGERLFYTL HLQVEKIENY KLNCEELSQR MLYLYEECGN SYI--

AtbHLH100 SHGERLFYSM HLQIK---NG QVNSEELGDR LLYLYEKCGH SFT--

AtbHLH101 SSTARIFYTL HLQMRG--DC KVRLEELING MLLGLRQS-- -----

CmbHLH101 SSNGSLFFTL LLQ------- ---------- ---------- -----

SlbHLH068 SFGDKIFHSL HFQMQG--PI EMDIQVLKTK LLVMCEKRRK NSYIV

**Supplementary Data S2.** Multiple alignment of fefe-bHLH38 with subgroup-Ib proteins in melon, arabidopsis and tomato. AtbHLH38/39/100/101 corresponds to subgroup-Ib proteins in arabidopsis, CmbHLH101 corresponds to bHLH101 in melon, SlbHLH068 corresponds to bHLH068 in tomato.
