# Supplementary material for: Mapping and Characterization of the fefe Gene That Controls Iron Uptake in Melon (Cucumis melo L.)
Source: Front Plant Sci. 2017 Jun 14;8:1003. doi: 10.3389/fpls.2017.01003 (PMC5470102; doi:10.3389/fpls.2017.01003)
Supplement: Supplementary file 3 [file Data_Sheet_3.DOCX]

Supplementary Material

**Mapping and characterization of the *fefe* gene that controls iron uptake in melon (*Cucumis melo*. L)**

Raghuprakash Kastoori Ramamurthy and Brian M. Waters

*** Correspondence:** Corresponding Author: Brian M. Waters ([bwaters2@unl.edu](mailto:bwaters2@unl.edu))

**Supplementary Data S3.** Genomic sequence information for *fefe-bHLH38* (sequence in italic fonts indicate LTR regions. Bold font indicate the 14 bp transposon footprint).

>*fefe* *bHLH38* Contig-genomic

GCACCACACAAAACAATTTCAAGTCACCAATATTTTCCAAAACCCTCTAGTTTCCTAATCCATTTTCATTTCTATCATGTTGGCAGTTTCCTCTCCTTTGTTTTCCCCTCATCAATGGCAATTGGAAGATCCCATCTCTCTCCATCACCAACACAACTCTCTCTTCTCTCCCTTTGAACCTTCAGATCATTCATTTTACCTCCAATTCCCTCCACCCCCGTTGGACCCTTCGCACGACCACTATCCTTCCTCTGCTGCGCCCTCCCCAGAAGCTGTCTCCAACGTCTCGAAGATGGCCAAGAAACTTAGCCATAATGCTAGTGAGCGCGATCGTCGAAAGAAGATCAACTCTCTTTACTCGTCTTTACGAGCCCTCCTTCCCTCCTCAGATCAAATGGTAAGGTTTACCATCTCTATTTAAAAAGTTAAAGTAATATCAAAACTTTAAGTTTAGTCCTTAACTATGTGATTAATTTTATAGACAAACCATACATGATCCAAATAATCAAACATATGTCAAACTCAAGTTAATTGGAATAACTTTACGTACCCTAGAAGTTGGAGAAATATATATAAATAAACATAAAAGTTAACCGTCCTTAATATCTTTTTCCTCTAGCCAAAATAGCATTGTGATTGTGGAATGGAGATTCAAATCTCTTGATTGTTATTTACTTTAATTTGTTAAATTATCCTCCTGTCGACTCCATTGTAGACTTTTAATCTCCATGGAGGTCCTTTGACCACCGTGAATAGTCGAAAACGTTCGTCCATCAATGATAAATTTAGTTGAAAGTCGTGGTCTTTTTTCTCACAGAAGAAACTGAGTAATCCGGCGACGATCTCGAGGATATTA

***TGAAGATTTTAAGA****AGGTATTGATTTAAAATAAAAGGATTGTTATTATTATTCTTTACAGAAGAGTATATATCTATTTATAATTGGCTTGTACAATTAGCCGTTAATCTATTCCTATAATTACGCTGGATGTTAAATATGGTATAATTGCAGCCTAATGCTTAATTGTAGCTAAATCCTATATATGTATACTGTATTAGCCGTTGACATGTTTCCATGTACAGCCTATGATGATTCTATGAATCTGAGGCCATTAATTCTGATCTTAATCCTTCAATA*

CTCCCCCTCAAGTTTGGTTCGAACAGGTTGTGAGCGCCTAACTTGCTGAGAATTTGTTGAGAGGTTTTCTTGTCCAAAGCTTTAGTGAAGATATCCGCTAGTTGTTCTTGGCTTCGGATGAAAGGAATCTCAATTTCCTTTGACTGCACTTTATCTCGTATAAAATGACAATCTACTTCTATGTGCTTTGTTCTCTCATGGAATACGGGGTTTGAAGCAATATGACGTGCCGCTTGGTTGTCACAGAACATTTGTATAGGTTCTGAACATTCAATTTGCATGTCGTGTAGCAGATGCTTGATCCAAATGAGTTCGCTTGCCGTTGATGCCATCGCTCTGTACTCAGCTTCTGCACTTGAACGAGCCACCACGTTCTGTTTCTTGCTCTTCCATGTTACTAGATTGCCACCTACGAAAGTGCAAAAACCAGTGGTTGATTTTCTGTCACAACTTCCGGCCCAATCTNGCATCAGAAAATCCAACCACAGTGTTAGTCGAGTTTTGTTTCATTAATATTCCTTGTCCAGGAGTGCCTTTGAGATATCTTAATATTCGGTTAATGGCTTCCATGTGACAAGTTCGAGGTGCGTGCATGAATTGGCTTACTATGCTAACTGCAAATGTAATATCAGGTCTAGTAACGGTTAGGTAGATTAATTTTCCAACTATCCTTTGATATTGGCNTTATGCCTGATAGTGGTTTACCATCCTCAGTGTTTAATTTGATGTTGGTTTCCATTGGACTAGTGGCTGGTTTGGTACCTAATTTACCAGTTTCTTTTAGTAGATCTAGTGTATACTTTCTTTGGGATAAAAACAGTCCTTTTGTCGAGTGTGCTATTTCTATTCCTAGAAAGTATGAGAGTTTACCTAGATCTTTTATGTCAAATTTTCTTTTTAACATTTCTTTGACTTCTTTTAATTTTTGGTTGTTATTACCGGAAATGATAATATCATCCACATAAACTAAAATAATAGTAATTGAGTCACCATTCTTTCTTATGAACACAGAGCAGTCTGCAGTGCTTTTCTTGAAATTATTTTCTGTGAGAAAAGTACTAAGTTTGGCATACCAAGCTCTTGGTGACTGTTTAAGGCCATAAATAGCCTTTCGAAGTTTGCATACCTTGGAGGTTTCGAAATACCCTGGTGGGGGTGTCATGTAAACTTCCTCCTCTAGATCTCCTTGTAAGAAAGCATTTTTTACATCCATTTGGAACAGATCCCACCCATGATTGGTTGCTACTGACATTAATATCCTAAAAGTGTTCATTTTTGCTACAGGGGCAAATGTTTCTTGATAGTCAATGCCGTATGTTTGGGTGAAACCTTTGGCAACTAATCTGGCTTTATACCTTTCAACTGTGCCATCACTGTTATATTTTATTTTATAGACCCATTTGCATCCTACTGGTTTCTTACCTTTGGGTAGTTCTACCATATCCCATGTGTTGTTTTGTTCTAAGGCTTTTAATTCTTCATTCATGGCTTGAATCCATATGGTCTGTTGTTTGGCCTCGTCATACGTGTTTGGTTCATTGTTACCGTCAAGTTTGCTTAAATAAATCTGGTAGGTTGGTGATACCTTGTTGTAATTAATAAAATTTTGGATAGGATACAAAACCTGATGGGATACAAAATCCCTTAACCTTGTTGAAGGTTGCCTTGTTCGTGTTGATCGTCGTCTGATTGTATCTTCTCCTTCTTCTTCTTGTCTGTCTTCTGTATTGTTCCGTTCATCCTCATAATCTCCCCCTGAAGAAGATGCGGAAGGATTTTCTTCATCGTCAAGGGAAGGAAAGAGGAATTGCAGAGTGCTTGGTGTTGCAGCGGTGGTGTCTTGTGTAGGCGTGAAGAATGGTTCATGCTCTCTGAAAACTACATCCCTGGAAATATACAGTTTATTTTGTTCGGGATCAAAGCACTTGTATCCCTTTTGGGTTGAGGAGTAGCCAAGAAAAATAGTTTTCACAGAGTTTTTATCTAGTTTGTCCTTTCGTTTTATATATACAAAGCAGGTGCATCCAAATACTCTAAGATGATCTAAGTCGATTTTTCTTCCTTTGAGAATTTCAAGAGGACTTAAATTATTGAGATTTGGGCTTGGTAATCTATTTATGATATAAGTAGCAGTTAGAATTGCATCTGACCAGAATTTTTTTGGAACATTATTCTGAAGTAGTAAAGCTCTTGTTTTTTCAAGAAGATGTCTGTTTTTTCTTTCAGAAACTCCATTTTGTTGTGGTGTATGAGTACATGTTGTTTGATGAAGAATACCATGTTGTTTGAAAAAATTGGTGAATTCCTTATTCACATACTCAGTACCATTGTCAGATCGAAAAATTTTGACTTGAGCATTGTATTGGTTGGTAATAAAATTAAAAAATTCTTGAAAGCATGAGAAGACTTCATTTTTGGTTTTTAAAAGATATACCCAAGTAGTTTTTGAAAAATCATCAATGAATGTAACATAGTATTTATAATGATTGTATGATTCTTCGGGAGAAGGTCCCCAAACATCAGAATGAATTAAATCAAAACATTTTTCTACTTTAGTTATGGAAGTAGGAAAAGGTAAACGAGTTTGTTTTGCAAATCTACAAGTATCACAACTAAAGGAATCATAATTATAATGAAAAAGTCTATTCAAAACCTGATCAGATGGATGGCCAAATCTTAAATGCAATAAATGTCCACGATCAGTGTTTTTACTTGATACAAAGCATTTATTATTTTGTTGAAGATAATATAGCCCGTTCCTAAGAATTCCTTCACCAATCATCTCCCCTGAGACTATGTCCTGAAAAATCACTTTTTCTGGTAAGAATATTACAGCACAATTAAGATCTTTGACAATCTTATTAACAGATAATAAATTAGAGTGAAAATCAGGTAGATACAAAACCTCATTGACTGGTTTGTTAAAAACAGAAATGGTTCCGGTACCGAAAATTTTGGTCTGACCACCATTAGCAGTTGTGACAAATTGTGGTTCATTGGAGGTTATCAAGTTTAAAAAATTGTTTGGACTACATGACATGTGATGCGTTGCTCCCGAATCAATAATCCAATTTGAGTCATGCTTATTCAAGTATATTGAATTATTTGAAAGTTTGAGTTCAGATAAACCTGAGTTCTGTTGCCTCGGCTGAAGTAGTTGATTTAATTGGTTAACCAGCTGCATGAGTTGATCCGAATTAGGTCCGGAAGGCCCAGCAGGCGTGACAAAGGAGATTGGGTCTTGCACTGGCCCAGCAGCAGTCGTTCCATAGAAGCCTGGTGGCCCGTGATCAGCTGGCCCTTGAAAGGGATTAGGGTTCATCCCATTCATCCCGAAGTCAGCCTTCGTATTTGTCCCGCTGTTGAGTTCTCCTATGGCGGAATGTGCCTCTCGGCGCCACCCGCCGTTGTTGGTACTTCCGCCACCTTTGCGTTGGGGCTTGAGATGCGGATGAAGAACCCAGCAACTCTCCTTGTTGTGACTCGATCGTTTGCAATGATCACACCACAAGTTCCCCTTTCCTCTGTCCTTGTTGTAGGTTGCGCGAAATGCTTGGTTGTCTGTTGATGGAGCTGGCGACGGATTCATGAGCCGTCTTCTTGATTCCTCCTGCTCAATCTTGAGAACTACATCATCAAACTGTGGCATTTCTGCCGATGAGAGAATTTGAGCCCGAATGGGTTCAAAACTGGAATCTAAAGCCCCGAGATAAGTATAAATTAATTCATGTTCGTTTCTTTTGTAAATTTCCTCTGGATTTATTGTTTCTGGGAGANTACATCTGTAACTCTTCCCATTTGGTCAGTATTTCGGCCACTAGGTCTGAGTTATTGAGGTTGCCTTGTTTGATGTTGGAAAGTTCTTGTTTTAATGAAAAAATATGTGCGAAGTTTTTTCCTTGACCATATCGGGTTTTGGCTTTTGTCCAGATTTCCTTTGAGGTTTTACAGTACATAAGAGCACTAGAGATTTTAGTGTCCATTGTGCTTAAGAGCCACGACATGATCATCTGATCTGTGGTCTCCCAATTTTCTATGGCTGTTAGTTCATCATCAGTAGGGTTGGCTTGGTCTTTTGGTTTTGGTTTTCCTTTGGATCCATTAATGAATGACCTTTTACCCTTACCCCCTAAGCCTATTTCCACAGATCTGGCCCATGGTACGTAGTTATTGTTACCAGTGAGGATATATGTGGTGATTTTGTTGTTTTCGATCATGTTAAGTTGCTGGTAGCAGAAATGTAATCCAGAGAGAAAAATAACAAAGAAAAGAACAGTTTTAAATTGATACTGATTTTGCTGATTTTTGCTGAGAAGAACAGATGCTTTCCTTGTTCCTTTCCACTGGTTGTCTCCCTCGGTTATGTTTTGCTCGATGAGGACAAATTTGAATTGATGCAGCAGAAGTTTCGTCACCTTTGTGGCGTTGAGTACTGCTTGCTAATGGCACCTTTGTGCTTGGTCTGAGAGCTTGAAATTGGGATCAAATTGCTCTGATACCA

***TGAAGATTTTAAGA***AGGTATTGATTTAAAATAAAAGGATTGTTATTATTATTCTTTACAGAAGAGTATATATCTATTTATAATTGGCTTGTACAATTAGCCGTTAATCTATTCCTATAATTACGCTGGATGTTAAATATGGTATAATTGCAGCCTAATGCTTAATTGTAGCTAAATCCTATATATGTATACTGTATTAGCCGTTGACATGTTTCCATGTACAGCCTATGATGATTCTATGAATCTGAGGCCATTAATTCTGATCTTAATCCTTCAATA

TATTAAGCTACATACCAGAGCTACAACAACAAGTGGAAGGGCAGATGAGGAAGAAAGAAGAGCTCATGGCAGCCATGGTTGGACAGGAAGTTAAGAATGATGAAGAAAAGAAAATGAAATCCGCAGCTTCGAGCTCCTCATCGATAATTTCTGCAAGTCGACTTAGTCGACATGAAATGGCGATTCAGATATCCACTGATATCAACGGCTGCCAGCGGAATTATTTGTCGGAAATATTATGTTGCTTGGAAGAAGAAGGTCTCCTTCTGTTGAATGCTTCTTCTTTTGAGTCTTTTGATGGGAAGGTTTTCAATCGAATTCCCGCGGCCGCCATGGCGGCCGGGAGCATGCGACGTCGGGCCCAATTCGCCCTATAGTGAGTCGTATTACAATTCACTGGCCGTCGTTTTACAACGTCGTGACTGGGAAAACCCTGGCGTTACCCAACTTAATCGCCTTGCAGCACATCCCCCTTTCGCCAGCTGGCGTAATAGCGAAGAGGCCCNACCGATCGCCCTTCCNACAGTTGCGNNGCCTGAATGG
