# Supplementary figures and images for: Mapping and Characterization of the fefe Gene That Controls Iron Uptake in Melon (Cucumis melo L.)
Source: Front Plant Sci. 2017 Jun 14;8:1003. doi: 10.3389/fpls.2017.01003 (PMC5470102; doi:10.3389/fpls.2017.01003)

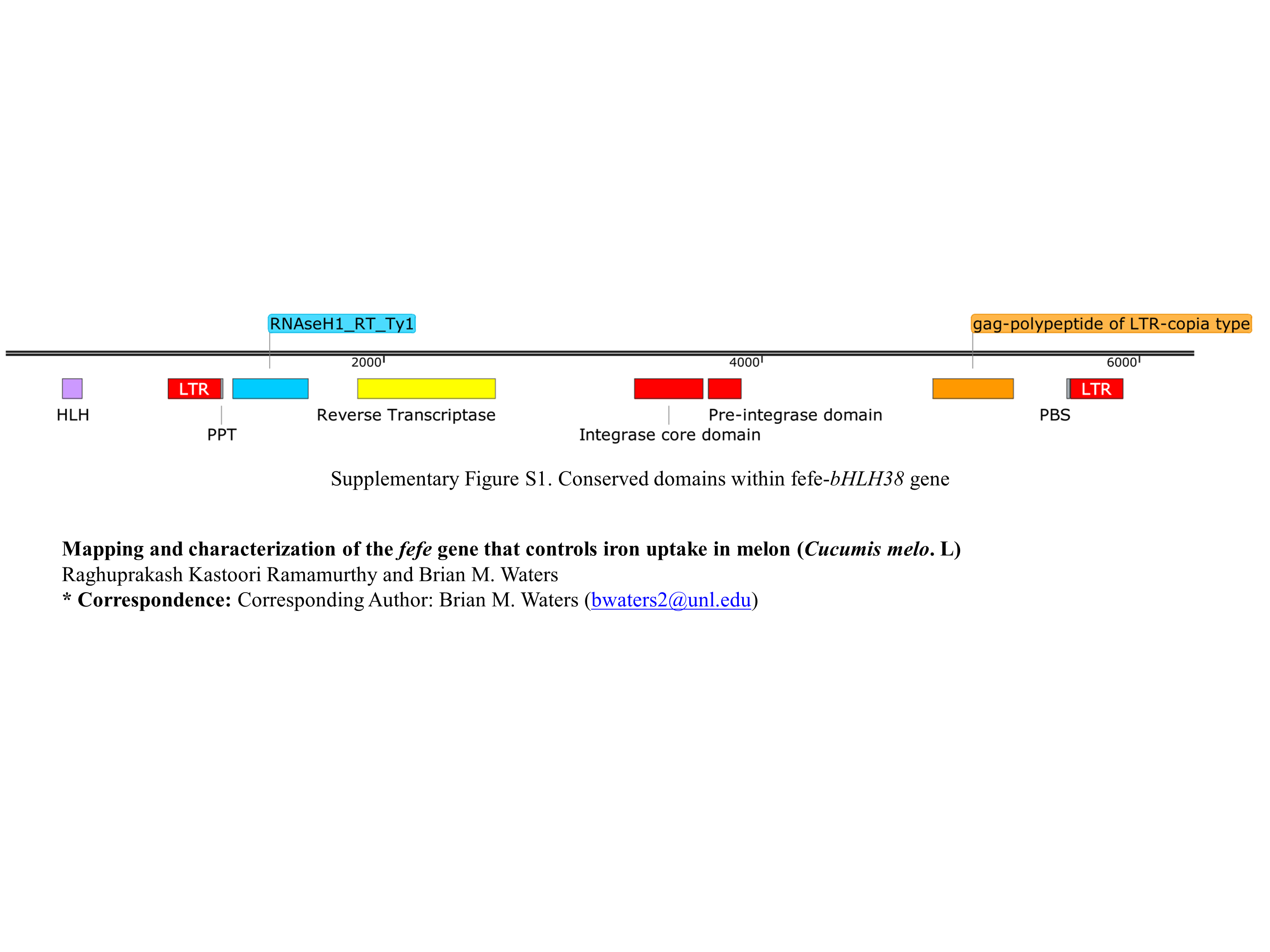

Supplement: Supplementary file 4 [file Image_1.TIF]

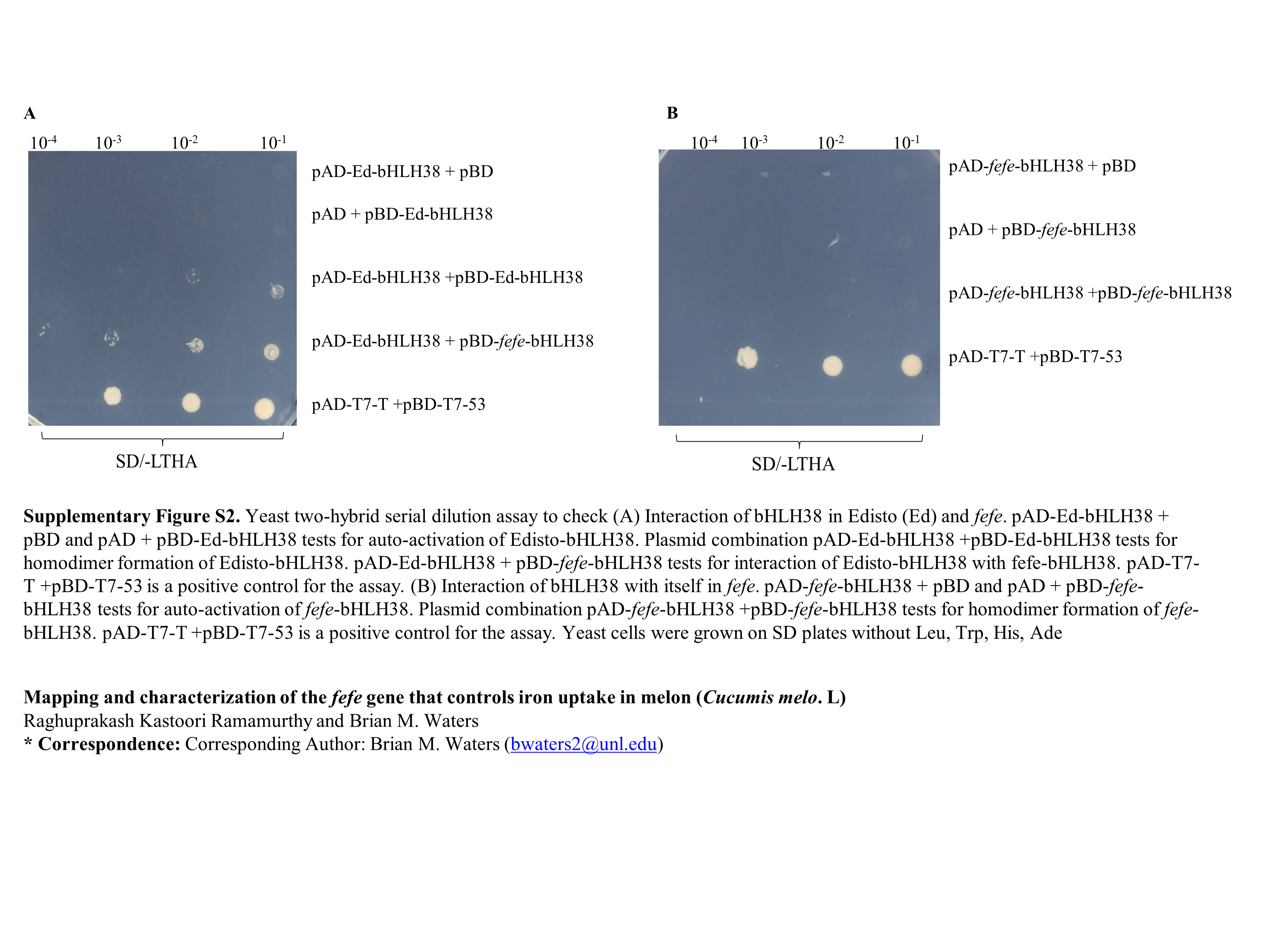

Supplement: Supplementary file 5 [file Image_2.TIF]
